# The Multidisciplinary Team (MDT) Approach and Quality of Care

**DOI:** 10.3389/fonc.2020.00085

**Published:** 2020-03-20

**Authors:** Miren Taberna, Francisco Gil Moncayo, Enric Jané-Salas, Maite Antonio, Lorena Arribas, Esther Vilajosana, Elisabet Peralvez Torres, Ricard Mesía

**Affiliations:** ^1^Medical Oncology Department, Catalan Institute of Oncology (ICO), ONCOBELL, IDIBELL, L'Hospitalet de Llobregat, Barcelona, Spain; ^2^Psicooncology Department, Catalan Institute of Oncology (ICO), IDIBELL, L'Hospitalet de Llobregat, Barcelona, Spain; ^3^Department of Odontostomatology, Faculty of Medicine and Health Sciences (Dentistry), University of Barcelona, Barcelona, Spain; ^4^Oral Health and Masticatory System Group (Bellvitge Biomedical Research Institute) IDIBELL, L'Hospitalet de Llobregat, University of Barcelona, Barcelona, Spain; ^5^Oncogeriatrics Unit, Catalan Institute of Oncology, L'Hospitalet de Llobregat, Barcelona, Spain; ^6^Clinical Nutrition Unit, Catalan Institute of Oncology (ICO), IDIBELL, L'Hospitalet de Llobregat, University of Barcelona, Barcelona, Spain; ^7^Head and Neck Nurse, Head and Neck Functional Unit, Catalan Institute of Oncology (ICO), L'Hospitalet de Llobregat, Barcelona, Spain; ^8^Expert SLP in Oncologic Patients, Head of SLP's Department, Atos Medical Spain, Barcelona, Spain; ^9^Medical Oncology Department, Catalan Institute of Oncology (ICO), B-ARGO, Barcelona, Spain

**Keywords:** head and neck cancer, head and cancer unit, multidisciplinary team, tumor board, quality of care

## Abstract

The core function of a multidisciplinary team (MDT) is to bring together a group of healthcare professionals from different fields in order to determine patients' treatment plan. Most of head and neck cancer (HNC) units are currently led by MDTs that at least include ENT and maxillofacial surgeons, radiation and medical oncologists. HNC often compromise relevant structures of the upper aerodigestive tract involving functions such as speech, swallowing and breathing, among others. The impairment of these functions can significantly impact patients' quality of life and psychosocial status, and highlights the crucial role of specialized nurses, dietitians, psycho-oncologists, social workers, and onco-geriatricians, among others. Hence, these professionals should be integrated in HNC MDTs. In addition, involving translational research teams should also be considered, as it will help reducing the existing gap between basic research and the daily clinical practice. The aim of this comprehensive review is to assess the role of the different supportive disciplines integrated in an MDT and how they help providing a better care to HNC patients during diagnosis, treatment and follow up.

## Introduction

A multidisciplinary team (MDT) in oncology is defined as the cooperation between different specialized professionals involved in cancer care with the overarching goal of improving treatment efficiency and patient care. Head and neck cancer (HNC) involves multiple and biologically distinct diseases that require different therapeutic approaches. Patient symptoms and treatment side-effects as well as physical and psychological impact will vary according to cancer location and treatment plan. Joining the efforts from different professionals is thought to improve patient management in contrast with the old idea of a global treatment offered by a single physician.

The multidisciplinary approach emerged in oncology in the mid-1980s, when the addition of chemotherapy to radiotherapy and/or surgery was proven to improve survival. In the meantime, organ-preservation strategies started to develop in HNC with the use of new available therapeutic techniques (1). The MDT initially consisted in a regulated committee that reviewed all new cancer patients and agreed on the therapeutic plan proposed by medical and radiation oncologist and surgical specialists based on their clinical expertise and the evidence available to date.

When the MDT members became aware that this approach was actually improving patient care, additional specialities focused on supportive interventions were included in the MDTs. The addition of the latter group of professionals improved the quality of cancer care by preventing and diminishing treatment side-effects, which in turn improved patient adherence and compliance to therapies (2). The natural evolution of this approach was the development of oncological functional units: disease-site specific cancers focused on the management and provision of services for cancer patients (3). These units integrate a multidisciplinary committee and include all the departments involved in a patient's care with the aim of facilitating the intervals and interactions between the different professionals, hence reducing time to diagnosis and/or commencement of treatment.

The first functional units created in Europe were the breast cancer treatment units. It was not until 1998 at the First European Breast Cancer Conference that many medical societies focused on breast cancer treatment claimed that breast cancer care, which includes diagnosis, treatment, genetic counseling, psycho-social support, and research, should be assembled in specialized units within an institution (4). This was captured by the European Society of Breast Cancer Specialist (EUSOMA) in the 2013 publication “the requirements of a specialist breast center,” a consensus on the minimum requirements for the multidisciplinary management of breast cancer in oncologic centers (5). These guidelines were well-received by many medical societies leading to the introduction of the multidisciplinary approach in many countries. To date, HNC MDTs have been successfully implemented in many countries and are now considered standard of care for the management of HNC patients (6). This comprehensive review evaluates the role of the different disciplines that should be integrated in MDTs and how they contribute to provide a better care to HNC patients during diagnosis, treatment and follow up.

## The Role of the HNC Specialized Clinical Nurse

Given its location, HNC often comes with a series of physical and functional complexities and as such, patients will require a comprehensive care at the bio-psycho-social level. Giving patients full support from the time of diagnosis will be crucial to complete the planned treatment. As an essential member of the MDT, the role of the specialized clinical nurse in this disease is to support patients during the whole diagnostic and treatment process, which will include not only performing nursing interventions (i.e., symptom, toxicity and/or wound management) but also operational case management such as treatment planning and coordination.

The nurse will facilitate and coordinate the activities among all the specialists of the MDT, framing their activities in care plans and integrating healthcare processes in collaboration with other professionals involved in cancer care. From a patient and family perspective, the nurse represents the anchor that will guarantee the continuity of care throughout the entire healthcare process, including the follow up.

At the time of diagnosis, the nurse will initially perform a comprehensive assessment of the patient and family (or primary caregivers). It is essential to establish a good relationship to involve both the patient and the family in the decision-making process and to educate them on how to prevent and manage treatment toxicity and how to identify new symptoms. This relationship is decisive to ensure patient adherence and compliance to treatment and will also optimize healthcare resources.

The role of the nurse specialist as part of the HNC MDT is focused on three areas of action:

Case management
The case management is described as the systematic effort to coordinate patient and family care in this complex pathology. Methodologically, the main goal is to achieve care results more efficiently, as it will allow a better control of professional resources ultimately impacting on health costs (7). The nurse applies the case management model in the context of a specific process or disease, in this case HNC patients within an hospital organization. One of the nurse aims is to work with and coordinate all the professionals that compose the MDT during the overall process of patient care. For the patient and family, the nurse represents the cornerstone from the diagnosis until the follow-up and has major supportive role until the resolution of acute toxicity. During the diagnosis and treatment planning, the nurse controls that the appointments schedule and required diagnostic assessments are carried out in a timely manner to avoid unnecessary delays in starting treatment. This is extremely important for those patients who undergo multimodal treatment, either concurrently or sequentially (i.e., definitive concurrent chemo-radiation, surgery followed by adjuvant radiation with/without chemotherapy). The nurse will not only ensure scheduling the treatments accordingly but will also help adapting it at every step based on each patient's requirements and needs.
  One of the most important roles of the nurse specialist is to offer urgent assistance to patients and families through a direct connection during daytime (i.e., a mobile phone that allows the patient/family to contact the nurse). This will allow the nurse to: resolve patient questions or concerns; help managing side-effects and symptoms; and screen for potentially severe problems that require urgent attention and/or reference to the emergency room. Moreover, it allows the treating physicians to be aware of any significant event, symptom or toxicity at all times and plan visits and/or modify treatments accordingly.
  The nurse must ensure that nursing care is incorporated into the design and implementation of clinical guidelines.
Operational role
  The nurse ensures that patient referrals from primary care or local specialists are scheduled in a timely manner to avoid delays, and links patients that require multimodality treatment with the physicians from the different departments (i.e., ENT surgical oncology, radiation oncology and/or medical oncology).
  Once the treatment decision has been made by the HNC multidisciplinary committee, the nurse schedules an appointment with the new patient and family/caregivers. During this first visit, a comprehensive assessment of the patient is conducted, which includes medical and psychosocial status as well as support requirements. The nurse facilitates information about the treatment, including plan and logistics, explains the toxicity and resolves all doubts or concerns that may appear. This visit is instrumental to consolidate the information previously given by the specialist physician (patient information is a key part of the quality of care).
  In addition to carrying out a comprehensive and individualized assessment, special attention is devoted to the detection of possible alterations that may hinder the usual treatment dynamics. This is of particular relevance in HNC patients. Specific circuits are activated to support the patients according to their needs: assessment of nutritional status, swallowing and phonation, dental evaluation, psychosocial support, rehabilitation, evaluation of toxic habits, oncogeriatrics, and palliative care. The nursing documentation for patient assessment is based on the proposal according to the Functional Health Patterns defined by Marjory Gordon (8).
Medical assistance
  To provide clinical assistance is a major key role of the HNC specialized clinical nurse. The nursing care in HNC is focused on the following areas:
  - Provide emotional support to patients and relatives after the impact of the diagnosis and during treatment.
  - Health education to patients and family members concerning prevention, early-detection and management of symptoms and side-effects, also providing tools to enhance their autonomy.
  - Collaboration in other healthcare areas (*i.e*. hospitalization, clinical trials).
  - Management of feeding tubes and gastrostomies.
  - Management of tracheostomies.
  - Post-surgical interventions, toxicity, and treatment-induced dermatitis management (i.e., radiotherapy, anti-EGFR antibodies).

## Dental Care For HNC Patients

Dental attention for HNC patients is essential and must be incorporated in each stage of the oncologic process. This process has different and independent stages where it is important to control the potential complications that can occur in the oral cavity after chemotherapy and radiotherapy (9–11). Potential toxicities and affected structures by chemotherapy, radiotherapy and/or surgery are summarized on Table 1.

**Table 1:** Potential outcomes of chemotherapy, radiotherapy and surgery and the affected structures implicated.

**Affected structures**	**Potential outcomes**
Skin radiation	Dermatitis
Oral mucosa	Mucositis Infections: fungal, viral, bacterial Pain
Teeth	Caries caused by hyposalivation or direct effect of RT
Jaws/bone	Osteoradionecrosis Mastication difficulties
Salivary glands	Hyposalivation/xerostomia
Muscles and soft tissues	Fibrosis and trismus Dysphagia Speech difficulties
Temporomandibular joint	Fibrosis and trismus
Tongue and taste buds	Taste dysfunction
Alteration of smell	Anosmia, cacosmia
Others	Pain Altered quality of life

*Modified from Villa and Akintoye (12)*.

Any dental procedure must be avoided within chemotherapy cycles due to the risk of complications. Chemotherapy-derived thrombocytopenia and neutropenia can lead to hemorrhage and infections: fungal (candidiasis), bacterial (periodontitis, abscess, necrotic gingivitis) and viral (herpes, cytomegalovirus) (13). Patients receiving chemotherapy, especially methotrexate, cyclophosphamide, cisplatin, and 5-fluorouracil, can suffer mucositis affecting the oral cavity health (14, 15). Preventive procedures to attenuate the severity of the chemotherapy-induced oral mucositis are: exhaustive oral hygiene, if possible, before the chemotherapy sessions; eating soft foods; do mouthwashes regularly when brushing the teeth (use a soft toothbrush) using saline solution (0.9%), sodium bicarbonate or methylcellulose; using floss in the inter-cycle stages of the chemotherapy to avoid bleeding and reducing the microbial load; and removal of prosthodontics (if removable) (12). Oral cryotherapy (30-min session) is a good preventive method in patients receiving 5-fluorouacil (12) and benzamine hydrochloride is a positive anti-inflammatory option. Low-level laser therapy (LLLT) or photobiomodulation has been used with good results based on the angiogenic effect, the stimulation on the production of serotonin, collagen and cortisol, and improving in conjunction the synthesis of nucleic acid (16).

Several agents can be recommended to reduce the severity of mucositis (i.e., oral Magic mouthwash^®^, a combination of antibiotic, antihistaminic or local anesthetic, antifungal, topical corticoid and a base that helps the other components to properly cover the affected mucosa). Dysgeusia is commonly observed in patients receiving chemotherapy, particularly cisplatin. It can decrease the appetite leading to reduced oral intake and weight loss. Zinc supplements have shown to be useful to improve the dysgeusia in a few studies (17–19).

Radiotherapy is the backbone of the multimodality treatment in HNC. Given the close location of HNC to vital anatomical structures, radiotherapy is often limited by the risk of toxicity to the surrounding organs at risk (20). The use of intensity modulated radiotherapy (IMRT), which adjusts the dose to the tumor's size, has significantly reduced but not completely eliminated the risk of late toxicity including xerostomia, cariës, trismus, and osteoradionecrosis (21). The evolution of upcoming radiation modalities such as proton-based radiotherapy with better selectivity of the tissue to be irradiated might help reducing further these toxicities (22, 23).

Preventive techniques to protect surrounding healthy tissue and reduce toxicity such as xerostomia, remain controversial. Some authors for example, prefer to use cytoprotective agents such as amifostine (WR2721), which avoid cellular oxidation in healthy cells (richer in alkaline phosphatase) preserving their correct function (24, 25).

Techniques to prevent irradiation of the salivary glands have been proposed. Auto-transplant of submandibular glands to submental space was proposed in 2001 as an effective, low-cost technique (26). Another proposed technique is the application of hydrogel in the submandibular gland, positioning it outside the irradiation area (27). Once xerostomia is established, the chances to reverse it or improve it significantly are low, and all the approaches are palliative, since none of them can regenerate the salivary glands, oral mucosa, muscular fibers, and dental tissue. A few palliative techniques have been proposed including acupuncture, low power laser, electrostimulation or the use of hyperbaric oxygen, although no randomized studies have proven their effectiveness (16, 28–30).

From a pharmacologic perspective, the administration of parasympathomimetic agents (pilocarpine, cevimeline, and bethanechol) has shown to improve the radiation-induced-xerostomia in the short-midterm but not in the long-term, and the side-effects can be a problem (31, 32). Alternative therapies such as herbs, traditional Chinese medicine and thyme honey have been also proposed in an attempt to improve patient quality of life (33, 34). In any case, continued oral hydration and the use of saliva substitutes (olive oil, betaine, or xylitol gum) are always advisable (35).

Patients undergoing oncologic treatments for HNC must take particular care of their oral health. Table 2 summarize dental assessment and interventions to be performed in HNC patients before, during and after treatment. Any dental treatment should be preventive, if possible, because any dental treatment after the oncologic treatments will be less effective (12, 36–38). Patients must follow a strict oral health routine, using remineralization elements such as fluoride. They must follow general advice such as maintaining a balanced diet and hydric-equilibrium. Patients, and those around them, must be aware of the acute and also long-term treatment-related side-effects, the potential functional and physical limitations they might encounter and the solutions we are currently able to provide. Continued research to reduce oral cavity toxicity and to allow the regeneration of damaged structures, in some cases irreversibly, is needed.

**Table 2:** Dental assessment and interventions to be performed in HNC patients before, during, and after treatment.

**Before**	**During**	**After**
Check the medical history carefully	Hydration, alkaline mouthwashes, oral mucosa protection.	Frequent control of the oral cavity and teeth (every 3 months)
Teach good oral hygiene habits	Control oral mucosa, with analgesia if needed	Good hydration. Saliva substitutes
Repair all possible teeth and remove compromised ones.	Bland diet Saliva substitutes	Parasympathomimetic Alternative therapies (low intensity laser, photobiomodulation, hyperbaric oxygen chamber, etc.)
Remove removable prosthodontics	Temporomandibular physiotherapy (this can be started before treatment)	After 6 months, consider oral rehabilitation
Explain treatment	Fluorine mouthwash without alcohol	Keep temporomandibular joint physiotherapy
Evaluate pre-treatment life quality	–	Evaluate the quality of life after the treatment
Fluorine supplementation	No dental intervention required	Fluorine mouthwash

## The Role Of the Specialized Dietitian

Nutritional management in HNC patients is particularly complex and face unique challenges as they are at high risk of malnutrition due to tumor location and treatment toxicities (39). Despite oncologic treatments have improved both locoregional control and survival (40, 41), the acute and long-term toxicities caused by these therapies still compromise dietary intake contributing to significant nutritional deterioration (42, 43).

The heterogeneity of symptoms in HNC patients due to tumor location often overlap with local toxicity, and can all interfere with oral intake. Hence, having the support of a specialized dietitian within the MDT is crucial to ensure that the patient's nutritional status is optimized (44, 45).

The prevalence of malnutrition at diagnosis can range from 42 to 77%, and it usually worsens during treatment (46, 47). Weight loss is one of the major concerns as more than the 85% of patients will lose a substantial amount of weight from diagnoses until up to 3 months after completing treatment (44). Early nutritional assessment to optimize patient's nutritional status and to evaluate the need of a prophylactic feeding tube can contribute to minimize the impact of acute toxicity, treatment interruptions and ultimately improve survival (48).

Many studies have reported the importance of screening malnutrition among cancer patients using different validated tools (49–51). However, given the high prevalence of malnutrition in HNC patients, an early complete nutritional assessment of all patients incorporating dietetic counseling even in those well-nourished seems to be more effective (44). Dietetic counseling ameliorates malnutrition and reduces unplanned hospital admissions during treatment (52). For those patients that are already malnourished, an early detection will help improve their nutritional status prior to treatment and will flag them to the treating physicians within the MDT to prioritize and/or adjust before the start of the oncological treatment,” A few studies have shown that an improvement in the nutritional support prior to treatment reduces the incidence of infections, the time of hospitalization and the severity of toxicity and leads to improved survival (53–56).

**Figure 1:**
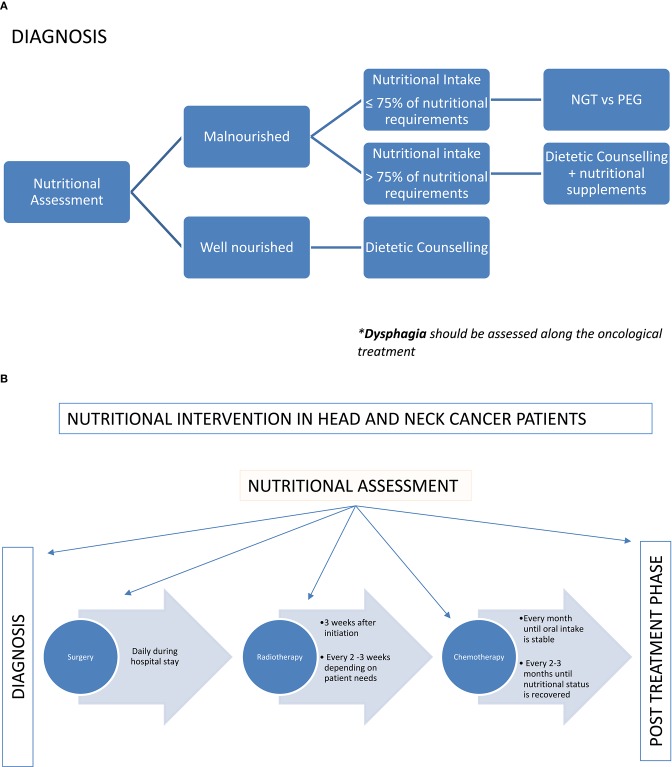
**(A)** Nutritional intervention in HNC patients according to the nutritional status. **(B)** Nutritional intervention in HNC patients according to the treatment plan.

Close nutritional monitoring during treatment is necessary to individualize and adjust the nutritional support to the emerging toxicity (47). At present, there is not enough evidence to support weekly instead of bi-weekly nutritional assessments during treatment (57) according to weight loss although weekly assessments seem to increase adherence to the nutritional interventions (58). Dietetic resources are usually limited; therefore, the coordination with other health professionals may help to improve the adherence to the nutritional support. Up to 38% of patients do not attend the follow-up appointments with the dietitian 4–8 weeks after treatment (57). Home visits, telemedicine or accommodating nutritional review right before or after the oncological evaluation may increase adherence to visits and ultimately enhance nutrition outcomes. Nutritional intervention in HNC patients is summarized in Figures 1A,B.

**Figure 2:**
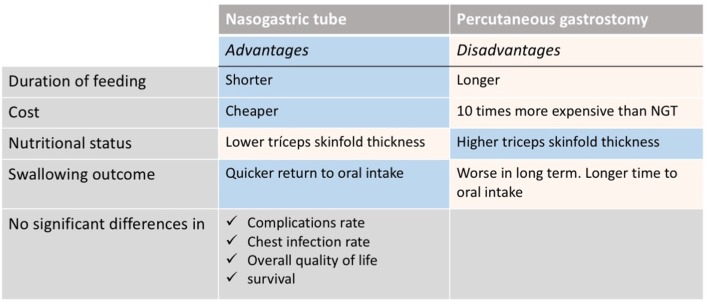
Nasogastric tube vs. percutaneous endoscopic gastrostomy: advantages and disadvantages [Extracted from Wang et al. (66)].

The nutritional intervention depends on multiple factors including tumor location and stage as well as type and intent of the oncological treatment (curative vs. palliative). In patients undergoing surgery, the nutritional intervention aims to meet the nutritional requirements right after the surgery trying to return to oral intake when possible. The use of gastrostomy feeding tube is still a matter of debate (59–63). Some studies have shown minimize weight loss and quick return to oral intake once the treatment is finished with the use of a nasogastric tube when clinically indicated (62, 64). However, other authors (61, 63, 65) report the use of prophylactic gastrostomy feeding tubes without increasing the risk of long-term swallowing dysfunction. Recently, a systematic review reported the advantages and disadvantages (Figure 2) of both percutaneous endoscopic gastrostomy and nasogastric feeding tubes (66). Patients that might require a prophylactic feeding tube prior treatment should be assessed by the dietitian at diagnosis. The indication should be individualized based on patient's baseline nutritional status, estimated time that feeding tube might be required and expected nutritional problems during treatment. Additionally, the capacity and characteristics of each hospital should be considered. The final recommendation should be agreed by both the patient and the MDT.

Sarcopenia, defined as low muscularity, has been recently shown to be a negative prognostic factor for overall survival in cancer patients (67). Specifically, in HNC, sarcopenia has been correlated with an increased rate of surgical complications in patients undergoing total laryngectomy (68) and with decreased overall survival in those treated with either radiotherapy alone (69) or with chemoradiotherapy (70). In addition to the nutritional support, the implementation of physical activity is also necessary to minimize nutritional and muscular deterioration in HNC patients. Some studies have integrated progressive resistance training programs during cancer treatment showing successful patient adherence and improvement in quality of life (71, 72).

Accumulating evidence suggests that up to 90% of HNC patients develop acute nutrition impairment due to the symptoms generated by the tumor location and treatment toxicity (i.e., dysphagia, mucositis…) (73, 74). Concurrent chemoradiotherapy is associated with higher rates of toxicity and complications when compared with surgery or radiation alone (74). Some of the treatment toxicities can be long-term and become chronic: swallowing dysfunction, xerostomia, dental problems, taste alterations, and weight loss have a significant impact on patient's quality of life (75, 76). In addition to the nutritional support, symptom-management (i.e., analgesia), psycho-oncological counseling, and speech and language rehabilitation therapy will be essential to improve their quality of life.

A few clinical guidelines for the nutritional management of HNC patients have been published (77, 78). These guidelines have significantly raised awareness on the impact of nutrition in HNC patients among oncologists and surgeons, increasing the number of early nutritional assessments and making it part of the treatment decision, particularly in patients with uncertain prognosis.

## Psychosocial Support in HNC Patients

The diagnosis of HNC is often accompanied by difficulties in eating, chewing, drinking, breathing, and speaking as well as by changes in physical appearance. All these alterations can lead to psychosocial dysfunction. There is evidence to suggest that psychosocial interventions in these patients are effective, and as such, clinical intervention programs should be developed as part of their cancer care.

### Psychosocial Needs

HNC patients experience profound functional and visible changes as a result of their disease and its treatment, having an important psychosocial impact on them and their families. The disease and treatment toxicity result in physical and psychological symptoms that patients must deal with, such as dysphagia and disfigurement. Patients have problems in the social and family setting, often related to the reduced ability to conduct many basic functions such as eating, speaking, and breathing. These problems can lead to limitations for work, and also for daily activities such as driving (79). Sometimes, it will be necessary to refer patients to a social worker to assist them with any financial burden and facilitate the access to the appropriate financial resources. Reduced libido and sexual enjoyment are common in individuals following surgery or chemoradiation. It is recommended that the treating physicians and health care professionals address this issue.

Cancer survivors with physical impairment or change in appearance or function have a high risk of psychosocial sequelae and diminished quality of life. This is especially common among patients with HNC, and it is important to help them coping with these psychosocial aspects into their routine cancer care throughout the illness trajectory.

### Psychological Distress

No one expects to be diagnosed of cancer. Patients have to assimilate and integrate the information about their condition and treatment options. HNC patients suffer from the visible nature of their disease but disfigurement and dysfunction can often also result from surgery and radiotherapy (79). As a consequence of these difficulties, patients can experience depression, social anxiety, reduced self-esteem, sexual difficulties, and a generalized sense of reduced quality of life (80, 81). Ineffective coping strategies such as helplessness, hopelessness, anxious preoccupation, and fatalism are strongly associated with anxiety and depression (82). Koster and Bergsma described HNC as more emotionally traumatic than any other type of cancer (83). After treatment, HNC patients were more distressed than other groups of patients. In term of coping, they had higher levels of anxious preoccupation than other cancer patients (84). Anxiety and depression is experienced by ~30–40% of patients following treatment of HNC (85, 86). One outcome of depression is suicide. Two head and neck cancer sites alone (tongue and pharynx) accounted for almost 20% of the total suicides among male patients with cancer (87).

### Psychological Interventions

Psychological interventions include: psychoeducational counseling, psychotherapy (individual), cognitive behavioral training, supportive, and group interventions. Social support is seen as an important factor in alleviating the emotional distress and social dysfunction experienced by patients with facial disfigurement. Education or psychoeducation is composed by information about the illness and treatment, coping strategies and communication skills. Counseling or emotional support is addressed to validate and normalize the emotional reactions, getting an adequate emotional well-being. Psychotherapy can be understood as a conversation process, with the objective to change the maladaptive narrative of patients, using different psychotherapeutic techniques. One of these psychotherapeutic approaches, the cognitive-behavioral therapy and medication can be considered to reduce anxious and depressive symptoms. The most recommended psychotherapeutic approach in cancer care is the integrative psychotherapy, which focused in promoting alternative narratives, helping to cope the illness and treatment. HNC support groups provide support to patients and families by giving them the opportunity to meet others in similar situations and learn that they are not alone (88).

The classic risk factors associated to HNC such as tobacco and alcohol habits need to be addressed. Similarly, the human papilloma virus (HPV) is a new risk factor, sexually transmitted, associated with a new profile of the HNC patient, with particular emotional needs.

In conclusion, psychosocial impact of head and neck cancer on patients and their families is significant. A psychological screening intervention is an effective way to identify patients and relatives who are at risk and might be interested in receiving psychosocial support, improving physical, and psychological outcomes.

## The Role of the ONCO-Geriatrician

Cancer is primarily an aging associated disease. As a result of the aging of the Western population and of a long lifetime exposure to tobacco, alcohol and other carcinogens, HNC in older patients is becoming a growing problem (89, 90). Nowadays, over a half of all newly diagnosed HNCs are in patients ≥65 years and it is estimated that in the next decade this will increase by more than 60% (89, 90). As this patient population is usually underrepresented in clinical trials, data on treatment efficacy and safety is scarce, and evidence-based guidelines are lacking (91). Due to misperceptions about reduced survival and higher risk of toxicity in older patients, oncologists are often reluctant to treat them with standard of care therapies, and patients that could potentially be cured end up receiving suboptimal treatment (92). In the overall HNC patient population, treating physicians have to decide the therapeutic strategy balancing the HNC-related risk of death against the potential survival benefit from treatment. In the elderly, oncologists should pay particular attention to the potential increased risk of treatment toxicity, their life expectancy irrespective of their cancer and also their values and goals (93, 94).

HNC in the elderly presents with a different clinical profile when compared to younger patients: it is more frequent in women; less associated with tobacco exposure and HPV; the most common primary site is oral cavity and larynx, and they usually present without nodal involvement (95). HNC are frequently diagnosed in a locoregionally-advanced stage, and require multimodality treatment including surgery, radiotherapy and chemotherapy. The significant acute and late toxicities that come with these therapies have an impact in the quality of life of patients hence pose an extra challenge when deciding the best treatment strategy in elderly patients with HNC (96).

**Figure 3:**
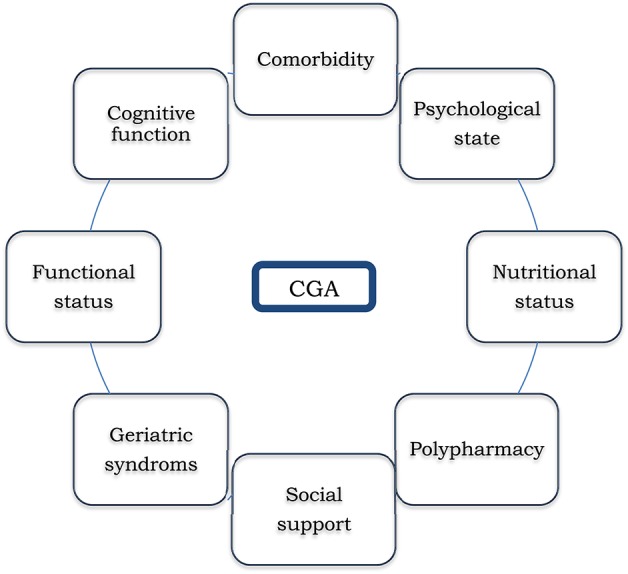
Domains evaluated in Comprehensive Geriatric Assessment (CGA).

Aging is a process that consists on a gradual loss of physiologic reserves that lead to impaired function ranging from robust health to frailty and, finally, to disability (97). Due to the heterogeneity of the aging process, chronological age can differ significantly from biological or functional age and treatment decisions should not be based solely on this value (98). Individuals who reach old age without loss of functional capacity or severe medical conditions should be able to receive the best therapeutic strategy according to their frailty profile. The main challenge is how to identify the right patients for the right treatment. Traditional tools used by oncologists to assess functional status, such as the Eastern Cooperative Group Performance Status (ECOG-PS) or Karnosky index (KI) have shown to be inaccurate in this population (99, 100). The Gold Standard to establish the frailty profile of the elderly is the Comprehensive Geriatric Assessment (CGA). Briefly, GCA is a multidimensional battery test that screens for impairment in all patients and includes factors that might impact on patient treatment tolerance and/or increased risk of toxicity: functional status, mood, cognition, social support, nutritional status, geriatric syndromes, polypharmacy, and comorbidity (101) (Figure 3).

**Figure 4:**
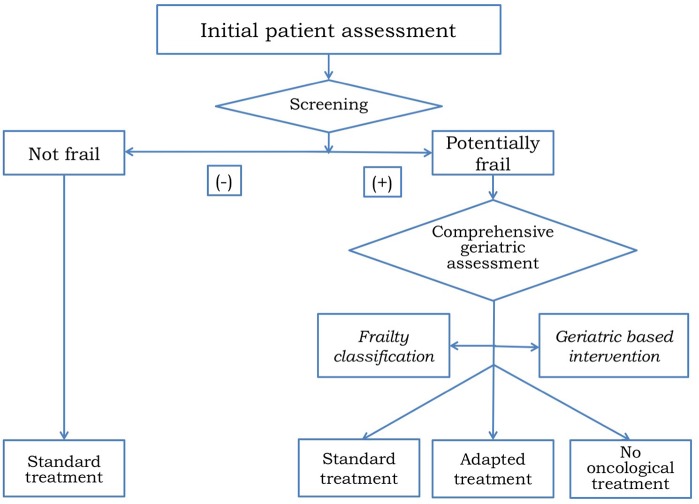
Algorithm for treatment decision making in older patients with HNC.

CGA helps to identify patients who are fit enough to receive standard of care treatment, those who are vulnerable and require an adapted treatment, and those unfit to receive any treatment and should be managed with best supportive care only (102, 103). In addition, CGA frequently detect geriatric impairments missed by routine oncological clinical assessment (104) which might be modifiable through subsequent geriatric-based interventions (105) (Figure 4). GCA has the ability to help decision-making by the initial frailty assessment but also can improve treatment adherence and tolerance by developing a tailored intervention and a supportive care plan during the follow-up. Since CGA requires an expert physician to interpret the results and therefore consumes time and resources, several screening tools like Vulnerable Elders Survey (VES-13) (106) or Geriatric-8 (G8) (107) have been developed to distinguish fit patients who are able to receive standard treatment from those who need a wider geriatric evaluation (108–110). Given the accumulating evidence supporting the role of CGA for clinical decision making, the main international societies and guidelines recommended CGA in patients aged 70 years or older before making a decision on cancer treatment (111–113). Most studies assessing the relevance of CGA have been done in cancer patients with different tumor types but the information among HNC is scarce (114). The on-going EGeSOR study is currently evaluating the impact of a multidimensional geriatric intervention based on CGA on outcome in elderly patients with HNC (NCT02025062) (115).

However, the optimal treatment should be decided considering both geriatric and oncological variables trying to find the proper balance between the tumor stage, the expected treatment benefit and toxicity and its potential impact on quality of life as well as the increased risk of toxicity and expected overall survival in the elderly. Based on this information, patients with a same geriatric profile might be considered fit enough to be treated with single-modality but not with a multi-modality treatment. That is why a CGA by an onco-geriatrician should be part of the HNC MDT (116). Recently, the ELAN-ONCOVAL (Elderly head and Neck Cancer-Oncology eValuation) study evaluating the use of a suited geriatric evaluation to stratify patients and guide therapy in patients ≤ 70 years old not amenable to surgery showed that, despite the initially planned treatment was changed only in 8% of the cases after a geriatric assessment, the number of patients requiring multidisciplinary interventions was significantly higher when the assessment was performed by geriatrician (71 vs. 51%) (117). The results of this study highlight the relevance of incorporating the onco-geriatrician in the decision making process for the elderly.

Multimodality treatment in HNC is associated with significant acute and long-term toxicity and represents an additional challenge in the elderly. Most of the studies evaluating therapy according to age in HNC patients did not show significant differences in survival between young and elderly patients (118). Nevertheless, older patients may experience greater treatment-related toxicity, specifically with higher intensity treatments (94). When evaluating the outcome of single modality treatments such as surgery or radiotherapy, no differences were seen between younger and older patients (119). Importantly, postsurgical complications, or toxicity rates were not influenced by chronological age. While the survival benefit from chemotherapy remained similar across age, older patients had higher rates of toxicity (nephrotoxicity, diarrhea, and thrombocytopenia) (119). The results regarding concomitant chemoradiotherapy are controversial, likely due to heterogeneous populations (120, 121). However, a study comparing elderly patients receiving radiotherapy alone vs. multimodality therapy, showed that patients from the multimodality group had similar survival rates to younger patients, while those treated with radiation alone had strikingly inferior outcome (122). The use of targeted therapies such as cetuximab is common in the elderly because they are perceived to be less toxic than platinum-based chemotherapy, but no prospective studies have compared the safety profiles in this specific patient population (91). As regards the use of immune-checkpoint inhibitors in the elderly, the trials conducted thus far do not seem to suggest that higher toxicity should be expected in this subgroup of patients (123). Based on this data, we can conclude that a subgroup of fit older patients can benefit from aggressive therapy, being comorbidity and functional age better predictors of treatment tolerance and efficacy rather than chronological age (124–126).

In summary, older patients with HNC require an “age-friendly” healthcare model based on “case management.” An individualized comprehensive assessment of the elderly patients leads to a more accurate treatment decision leading to a more efficient use of the healthcare resources. A specialized geriatric assessment of elderly HNC patients is crucial to drive the optimal oncological treatment strategy and as such, it should be integrated within the MDT.

## The Role of the Speech and Language Pathology Expert

Patients who have undergone a total laryngectomy (TL) will inevitably have to face a wide range of sequelae that will have a considerable impact on their quality of life.

From the speech and language pathology (SLP) perspective, the most obvious and limiting handicap is the loss of the laryngeal voice and, consequently, of the person's ability to communicate orally in the same way as they did until the moment of the surgery. The voice, in addition to the basic instrument for human communication, is for everyone a hallmark of self-identity (127) and reveals aspects such as personality traits, mood or gender.

Inability to speak, or to do it with a “new voice” totally different from the one that has been “the own voice” during a person's life, involves very important changes in that person's daily life, and can significantly impact on his/her social and familiar relationships, ultimately leading to anxiety, depression, and alterations in self-esteem and self-image (128).

### Vocal Rehabilitation

Vocal rehabilitation in patients treated with a TL to restore oral communication requires a process of adaptation and readjustment to this new voice that is crucial in order to improve and restore their quality of life (129). After the surgery, patient can be taught laryngeal techniques by an SLP expert through an intensive and structured work. The alternatives of communication after the TL, are:

- Esophageal speech

TL results in a permanent separation of the airway and digestive tract. In the absence of the larynx, a patient can learn to generate a new sound—laryngeal voice- through the vibration generated by the air passing through the pharyngo-esophageal segment. This fundamental frequency will be articulated in the oral cavity, generating different phonemes or speech sounds.

In the esophageal speech, the air is injected or inhaled from the oral or nasal cavity, to the upper esophageal sphincter, and immediately returned to the oral articulatory structures. The learning process of the esophageal speech can be long and requires an intensive, guided and structured work. Hence setting realistic goals in the short term will be important to prevent demotivation. It is also necessary to systematically evaluate patient's progress during the therapy in order to identify difficulties that could make this strategy impossible, and adapt it to patient possibilities.

- Tracheoesophageal speech (voice prosthesis)

The tracheoesophaigeal speech is obtained after a surgical procedure which consists of the generation of a fistula in the posterior wall of the trachea, where a voice prosthesis is placed. A voice prosthesis (VP) is a cylindrical, silicone device that has a one-way valve mechanism that allows the passage of expiratory air flow from the trachea to the neoglottis. Airflow through the esophageal structure will generate a new fundamental tone that, as with the esophageal voice, will later be converted into speech sounds. The use of a voice prosthesis for communication requires daily maintenance and needs to be replaced approximately every 6 months, (130–132) The rate of complications can range from 15 to 72% (132), being the most frequent the leak through de voice prosthesis and the leak around the tracheoesophageal prosthesis.

The involvement of the speech pathologist to train how to manage these complications is important in order to prevent and to minimize their impact, and is also required to provide a continuous follow-up in the rehabilitation process until stabilization. A proper follow-up by an SLP expert of patients with phonatory prostheses is associated with better overall results in their rehabilitation (133).

Compared with esophageal speech, tracheoesophageal speech has demonstrated better results in some voice analysis parameters such as fundamental frequency, maximum phonation time, intensity and other aspects as speech intelligibility (134). Some studies found a correlation between the use of a VP, with a positive restoration of quality of life, self-esteem, and sexual function, with the consequent decrease in the symptoms of depression and anxiety (135).

- Electrolarynx

The electrolarynx is an external device with a sound-head cap that, when activated and in contact with the skin, generates a vibration, usually monotonal, which is transmitted to the oral cavity. To speak with an electrolarynx, the patient has to press a button and move his mouth to articulate different sounds. This system can be very useful to facilitate immediate communication during the hospitalization period, as communication through esophageal speech or VP is not possible as it requires training. When voice alternatives fail, electrolarynx is an excellent solution to allow oral communication.

**Figure 5:**
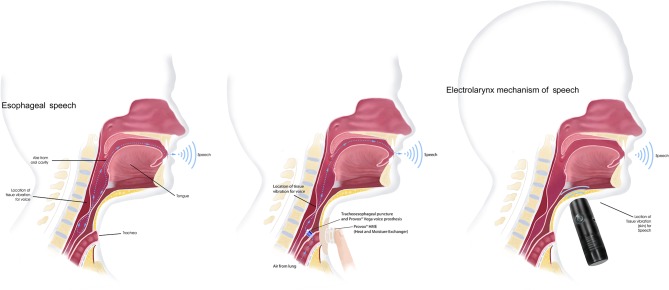
Voice after a total laryngectomy. Vocal rehabilitation alternatives after total removal of the larynx Images courtesy of Atos medical.

Overall, the best communication system is the one that will provide the best results according to patient communicative needs and expectations. Ideally, the laryngectomized patient could develop more than one communication strategy, such as a combination of an esophageal voice and the tracheoesophageal voice, as they can easily coexist (Figure 5). This will allow the patient to choose the most appropriate option to speak depending on the communicative context. Table 3 summarizes pre and post-surgical SLP recommended interventions.

**Table 3:** Areas of focus in SLP interventions.

**Pre-surgery phase**	
Goals	Solve all doubts that may arise to the patient and their relatives, related to what their life will be like, from the anatomical, physiological and functional point of view, after TL.
	Provide information regarding potential sequelae, such as vocal, pulmonary, eating and olfaction sequelaes. Information about their treatment and the existing devices to reduce the impact and preserve their quality of life, such as cannulas, adhesives, free hands devices for speech among others.
	Explain and show communication alternatives available.
	Promote contact with another person who has already been rehabilitated or, when appropriate, provide audiovisual materials with real examples of similar cases of patients that satisfactorily overcome their illness and rehabilitation process.
	Evaluation of vocal rehabilitation possibilities of the patient.
	Evaluate phonatory, respiratory, swallowing and olfactory patterns, to adjust an adequate prophylactic program if necessary, and determine realistic goals for rehabilitation according to the needs, expectations and commitment of the patient.
	Design a prophylactic program of pre-surgical exercises, according to time, treatment, and patient feasibility.
	Structure the therapeutic work plan after surgery, agreed between the patient and their SLP.
**Post-surgery phase**	
Goals	Fitting the proper system to help the patient for communication during hospitalization.
	Global evaluation to determine how is the starting point for rehabilitation, in terms of mood, communicative intention, scarring, fistulas, nasogastric tube, dysphagia, skin condition, configuration of the stoma, volume and characteristics of the secretions, voice prosthesis, weight, muscle tone…
	First adaptation of the rehabilitation devices.
	Review and start of the therapeutic work plan established in the pre-operative evaluation.
**Follow-up phase**	
Goals	Permanent review of concepts and doubts that the patient and or family may have.
	Prevention of difficulties associated with the use of rehabilitation devices through training in the proper use of it, and the understanding of the warning signs that the patient should inform to the professional.
	Promotion of patients' self-care regarding rehabilitation and management of the rehabilitation devices.
	Checking of the evolution of the established therapeutic goals.
	Upon discharge, providing the patient with an easily accessible SLP contact.

*Intervention of the expert SLP on main sequelae groups: voice, pulmonary, eating and olfaction. TL, total laryngectomy; SLP, speech and language pathologist'*.

In order to guide and individualize the rehabilitation therapy, many variables need to be considered: the type of surgery, adjuvant treatments, patient psycho-social status and cultural environment, motor and visual skills, communicative needs, and patient's motivation. Furthermore, speech therapy protocols should not be developed in isolation. The accumulated evidence to date and the available clinical guidelines support the idea that a multidisciplinary approach of a patient with a TL, leads to a greater and better overall therapeutic success, and facilitates the restoration of an optimal level of quality of life (129). Thus, the work of the SLP should be integrated into a well-structured MDT that guarantee a good information flow among professionals who are specialists in the different related areas (136).

In addition to the diagnostic impact, HNC patients must face the concern on how their life might change after treatment. Research and clinical experience highlight the benefits of an early involvement of an SLP expert in the treatment of TL patients who suffer voice alteration, as well as a longitudinal follow up until rehabilitation discharge.

Providing information, support, and solving issues related to sequelae will facilitate a better quality of care and greater therapeutic success, which in turn will have a positive impact on the primary purpose of the multidisciplinary approach; restore as much as possible, the quality of life of our HNC patients.

## The Clinical and Translational Research in HNC Patients

Head and neck MDT and HNC units have been shown to be an effective tool to facilitate collaboration between professionals and hence improve care outcomes. This concept is accepted worldwide as the “gold standard” of cancer care (137).

Although MDTs are the central component of cancer care in many countries, there is a notable gap regarding how clinical and translational research can be integrated into these teams. Several publications involving multiple cancer types have described the importance of MDTs and oncological functional units to facilitate the smooth cooperation between cancer care professionals and improve patient cancer care. However, none of them discussed the potential benefits of incorporating the translational research teams within these units (6, 138).

We believe that clinical and translational research should be integrated within the HNC units. The prognosis of HNC patients remains guarded with a 5-year survival rate of 50% (139). The implementation of clinical trials provides an opportunity to offer more effective and less toxic treatment options in these patients. As HNC management is multidisciplinary, involving the different MDT professionals in the development of clinical trials is essential to design more accurate studies, especially in the locoregionally-advanced setting. The management of recurrent/metastatic disease is often complex and also requires multidisciplinary evaluation. Adapting the diagnostic and treatment circuits to facilitate the screening assessments required for clinical trials in this setting such as biopsies will avoid delays in the commencement of treatment and the successful patient enrolment in these studies.

Additionally, the integration of translational research teams in tumor boards and MDTs can help reducing the existing gap between current clinical practice and basic research. The input that basic researchers can receive from clinicians might be useful to guide translational projects. The reciprocal interaction and feedback from researchers and physicians will also contribute to improve prospective studies and determine the feasibility of the correlative analysis. The success of a translational project involving patient care is based on the coordination of the team. Tumor biopsies and collection of other specimens such as saliva, blood or stool are currently requested by prospective clinical and also correlative studies. Therefore, the collaboration and the timely coordination between research laboratories and clinics are crucial to conduct these studies smoothly and successfully.

Proposed recommendations to succeed in the integration of clinical and translational research within the MDT and HNC units are:

- Elect a coordinator for clinical and translational research within the unit.
- Promote periodic meetings to update projects and explain novel proposals.
- Make it open to all members of the unit so that everyone can contribute with new ideas and lead projects.
- Include educational programs to young members and trainees.
- Create working groups to distribute projects with appointed project leaders among the MDT members.

## Conclusion

MDTs and oncologic functional units significantly improve the quality of cancer care. The integration of all the departments and professionals involved in the treatment of a specific cancer guarantees full and continued support to patients during diagnosis, treatment and follow-up periods and it is perceived positively by most patients. The different members of an MDT will provide close management of symptoms and acute/long-term side effects; adequate nutritional support, psychosocial reinforcement, and individualized follow-up. A comprehensive assessment and monitoring of HNC patients by specialized MDTs will result in better treatment adherence and tolerance, reduction in long-term side effects, improved quality of life and ultimately improved treatment outcome and survival.

## Author Contributions

MT and RM: review concept, review design, and interpretation. All authors: manuscript preparation and manuscript review.

### Conflict of Interest

RM has received personal fees and non-financial support from Merck, and personal fees from AstraZeneca, Merck, Bristol Myers, Nanobiotix and MSD. MT has received non-financial support from Merck and Astra Zeneca, and personal fees from Merck, Nanobiotics, MSD and Bristol Myers. Medical oncology department has received sponsorship for grants from Merck. MA has received personal fess from Rovi, Aspen and Novartis. LA has received personal fees and non-financial support from Nutricia, Nestle Health Science and Merck. The remaining authors declare that the research was conducted in the absence of any commercial or financial relationships that could be construed as a potential conflict of interest.
